# The Contribution of Innate Immunity in Large-Vessel Vasculitis: Detangling New Pathomechanisms beyond the Onset of Vascular Inflammation

**DOI:** 10.3390/cells13030271

**Published:** 2024-02-01

**Authors:** Lidia La Barbera, Chiara Rizzo, Federica Camarda, Giuseppe Miceli, Antonino Tuttolomondo, Giuliana Guggino

**Affiliations:** 1 Department of Health Promotion, Mother and Child Care, Internal Medicine and Medical Specialties, Rheumatology Section, University of Palermo, 90133 Palermo, Italy; lidialb90@gmail.com (L.L.B.); chiararizzo87@gmail.com (C.R.); federicacamarda@gmail.com (F.C.); 2 Unit of Internal Medicine and Stroke, Department of Health Promotion, Maternal and Child Care, Internal Medicine and Specialized Medicine, University of Palermo, 90133 Palermo, Italy; miceli.gpp@gmail.com (G.M.); bruno.tuttolomondo@unipa.it (A.T.)

**Keywords:** giant cell arteritis, takayasu arteritis, large vessel vasculitis, innate immunity, vascular inflammation

## Abstract

Large-vessel vasculitis (LVV) are autoimmune and autoinflammatory diseases focused on vascular inflammation. The central core of the intricate immunological and molecular network resides in the disruption of the “privileged immune state” of the arterial wall. The outbreak, initially primed by dendritic cells (DC), is then continuously powered in a feed-forward loop by the intimate cooperation between innate and adaptive immunity. If the role of adaptive immunity has been largely elucidated, knowledge of the critical function of innate immunity in LVV is still fragile. A growing body of evidence has strengthened the active role of innate immunity players and their key signaling pathways in orchestrating the complex pathomechanisms underlying LVV. Besides DC, macrophages are crucial culprits in LVV development and participate across all phases of vascular inflammation, culminating in vessel wall remodeling. In recent years, the variety of potential pathogenic actors has expanded to include neutrophils, mast cells, and soluble mediators, including the complement system. Interestingly, new insights have recently linked the inflammasome to vascular inflammation, paving the way for its potential pathogenic role in LVV. Overall, these observations encourage a new conceptual approach that includes a more in-depth study of innate immunity pathways in LVV to guide future targeted therapies.

## 1. Introduction

Large-vessel vasculitis (LVV) are granulomatous vasculitis affecting medium- and large-sized arteries, especially the aorta and its main branches [1]. They are mainly represented by giant cell arteritis (GCA) and Takayasu arteritis (TAK), both marked by vessel wall inflammation and remodeling, accounting for arterial stenosis and subsequent ischemic manifestations or aortic aneurysms and dissection [2]. Such dangerous conditions make LVV potentially life-threatening disease because of the broad umbrella of complications associated with high mortality and high rate of recurrence [3]. Although the two entities share similar immunopathologic features and vascular wall damage, they diverge in geographic distribution and age of disease onset.

GCA affects adults older than 50 years, and its incidence follows a north-to-south gradient, while TAK is more prevalent in young Asian women [4,5]. Thus, age is a major contributor to the development of GCA, as explained by the changes that immune cells undergo during the aging process [6]. In addition to the decline in naïve T and T regulatory (Treg) cells, release of pro-inflammatory cytokines, displacement of CD4 and CD8 cell trafficking, and narrowing of T-cell receptor diversity, immunosenescence also profoundly affects the innate immune system [7,8]. The main alterations described concern impaired phagocytosis by macrophages and polymorphonuclear (PMN) leukocytes and decreased antigen-presenting ability by dendritic cells (DC), in a context of systemic inflammation dominated mainly by tumor necrosis factor alpha (TNF-α) and interleukin (IL)-6 [9,10,11]. The aging process also affects the architecture of arterial tissue through medial degeneration, calcium deposition, wall thickening, and increased stiffness. Collectively, all these factors contribute to the occurrence of vasculitis.

LVV are HLA-associated diseases, which is highly suggestive of a pivotal function of T cells and antigen recognition in the immunopathogenic mechanisms of such disorders [12]. Thereby, the onset of vascular inflammation coincides with the disruption of the “immune privileged state” of the arterial wall. The authors of this pivotal phase are the DC, primarily responsible for the loss of tolerance and the shaping of innate and adaptive immunity. In LVV, adventitia and vasa vasorum are the initial sites where inflammation starts and then extends to the remaining layers [13]. In this setting, the innate system participates critically in the origins of inflammatory lesions through cross-linking with T cells and the release of effector cytokines, chemokines, and growth factors, ensuing the elicitation of a maladaptive immune response [14]. 

This is reflected in the skew of CD4^+^ T cells toward effector cells, with an increased frequency of IFN-γ^+^, T helper (Th)1 cells, IL-17^+^ Th17 cells, and a reduction in Treg [15,16,17].

If DC are drivers of the dysregulated immune response within the arterial wall, another remarkable cell subset that is critically involved in all stages of the vascular inflammatory process is represented by macrophages. The latter intervene both in the early phase and in the advanced phase of the disease, where they take part, assisted by relevant molecules, in vascular remodeling [18].

The current paradigm has been further expanded by the latest evidence on the role of immune checkpoint ligand and receptor interactions between T cells and antigen-presenting cells (APCs) in LVV. 

In healthy arteries, immune checkpoints provide the negative signals needed to prevent T-cell activation and dampen inflammation. This protective mechanism is shattered in GCA, where the inflammatory process is overpowered in a feed-forward loop [19].

Emphasizing the relevance of innate components of the immune system, besides arterial wall DC and macrophages, literature data describe an important role for neutrophils, mast cells, and soluble mediators, including the complement system [20]. Interestingly, new insights have recently linked the inflammasome to vascular inflammation, paving the way for its potential pathogenic role in LVV. 

The interplay between innate and adaptive immunity is of paramount importance in LVV and acts at every stage of the pathogenic process. The innate system contributes profoundly to the commitment of T cells toward a vasculitogenic phenotype that elicits loss of tolerance and subsequent persistent active inflammation in the vessel wall [12].

Hence, while the role of adaptive immunity has been extensively clarified, knowledge about the core function of innate immunity in LVV is still fragile and lacking.

The purpose of this review is to highlight recent progress in understanding the particular contribution of innate immunity in LVV, providing a broader perspective on the altered signaling pathways in which these cell subsets are involved (Table 1). This may lay the groundwork for future studies that address critical topics, especially regarding the potential therapeutic implications of tackling innate immunity in LVV. 

## 2. Dendritic Cells Drive the Dysregulated Immune Response within the Arterial Wall 

Human arteries possess tissue-resident DC, the so-called vascular (vas)DC, bordering adventitia and media layers. In LVV, DC residing in the adventitia have a critical role in initiating vascular inflammation, directing the breakdown of self-tolerance, and promoting abnormal activation of T cells [21]. Specifically, once activated by danger signals detected via the toll-like receptor (TLR), DC undergo functional modifications that mainly consist in the production of cytokines and chemokines and the expression of high levels of the class-II major histocompatibility complex (MHC-II) and co-stimulatory molecules (CD80 and CD86), responsible for the recruitment and activation of CD4^+^ T cells in the arterial wall [22]. Furthermore, DC are more abundant in GCA than in healthy arteries, suggesting their massive influx to the vessel wall during active disease, where they are retained due to the expression of CCR7 and the high local production of its cognate ligands (CCL19 and CCL21). 

TLR frontline the body’s defense by expressing pattern recognition receptors recognizing pathogen-associated molecular patterns (PAMPs) or danger-associated molecular patterns (DAMPs), which, once activated, promote the triggering of nuclear factor kappa-light-chain-enhancer of activated B cells (NF-kB) [12]. However, their relevance in LVV goes beyond the early stage of the disease process. Indeed, the TLR profiles expressed by vasDC are unique to each vascular territory and are instrumental in shaping the architecture of vasculitis [23]. In this regard, in GCA, it has been demonstrated that TLR4 ligands trigger Th17 recruitment, leading to transmural arteritis; conversely, TLR5 ligands promote adventitial vasculitis close to the vasa vasorum [24,25].

TLR-mediated activation of vasculitogenic DC could further boost the theory of the interaction between a potential infection and the initiation of GCA. Thus, infection-derived PAMPs could trigger adventitial DC through activation of TLR, starting, in turn, the downstream inflammatory cascade [26]. Despite these suggestive clues, no specific causative microorganism has been identified so far, and recent studies focusing on the microbiota of temporal specimens or thoracic aortic aneurysms in GCA have yielded inconsistent findings [23,27].

Activated DC express chemokines (CCL18, CCL19, and CCL21) implicated in their own maturation and recruitment of naïve T cells and produce cytokines (IL-6, IL-18, IL-23, IL-32, and IL-33) that enhance the GCA systemic inflammatory framework [28].

In GCA, DC are more inclined to T cell activation as a result of a defect in the co-inhibitory signal mediated by the programmed cell death protein (PD)-1/PD-ligand (PD-L)1 immune checkpoint expression [29]. PD-L1 and PD-L2, expressed by APC, bind PD-1 localized on T cells, inducing their apoptosis, anergy, IL-10 production, and polarization into Treg. Notably, the immunoprotective role of this axis is necessary for the immune homeostasis of the arterial wall, which is instead disrupted in GCA, as further confirmed by the increased risk of drug-induced vasculitis in patients under checkpoint inhibitors [30,31,32,33]. 

Recently, it has been described a decreased PD-L1 expression on vasDC in GCA coupled with the aberrant activation of PD-1^+^ T cells and the production of interferon (IFN)-γ, IL-17, and IL-21. To assess the functional activity of PD-1^+^ T cells in the inflamed vessel wall, a model system of GCA created by engrafting normal human arteries into immunocompromised mice that developed vasculitis was used. Treatment of human artery-SCID chimeras with anti-PD-1 antibodies resulted in increased PD-1^+^ T cell infiltration associated with intramural neoangiogenesis and intima hyperplasia [34].

The role of DC as a promoter of vascular inflammation is also suggested in TAK, where T cells co-localize with DC in the adventitia of aortic specimens along with evidence of moderate to strong expression of costimulatory molecules, such as B7-1, B7-2, CD40, CD27L, CD30L, and OX40L in smooth muscle and interstitial cells in the inflammatory lesions of the media [35,36]. Unlike GCA, the involvement of DC in TAK remains marginal compared with the core function of T lymphocytes in the pathophysiology of the disease, as confirmed by the potential benefit of T lymphocyte-targeted therapies. This is supported by a recent study in which fascin+ DC were significantly more abundant in the intima in GCA than in TAK [37]. Since fascin is a specific marker only for mature antigen-presenting DC, these findings indicate that antigen presentation in the intima is relatively lower in TAK than in GCA [37]. 

## 3. Macrophages Are Critically Involved across All Phases of Vascular Inflammation 

Monocytes are precursors of tissue macrophages, resident phagocytic cells implicated in host defense and tissue homeostasis. At the same time, macrophages are crucial culprits in LVV development and, together with T lymphocytes, participate in granuloma formation as the hallmark of disease [38].

Two subsets of monocytes have been identified depending on their phenotype, function, and chemotaxis pathway: classical monocytes (CD14brightCD16neg), recruited mainly via the CCR2-CCL2 axis, and non-classical monocytes (CD14dimCD16+), recruited via the CX3CR1-CX3CL1 axis. Both classical and non-classical monocytes were found in the temporal artery biopsies of GCA patients, suggesting that further studies are needed to better characterize their different roles in vascular inflammation [39].

In parallel, two main types of macrophages have been described, polarized in response to the vascular cytokine milieu: M1 and M2 phenotypes, reflecting Th1 and Th2 responses, respectively.

In GCA, activated M1 macrophages reside both in the adventitia, where they produce proinflammatory cytokines (IL-1 and IL-6) and in the media layer, where they promote arterial wall degradation by releasing matrix metalloproteinase (MMP) and reactive oxygen species (ROS). M2-activated macrophages reside at the media-intima junction, and through dysregulated repair processes and vascular endothelial growth factor (VEGF) production, they mediate wall thickening and the occurrence of stenotic lesions [40].

In the aorta of TAK patients, macrophages are the most frequent cell type, followed by T, B, and NK cells, and are found in all layers of the vessel. A recent study analyzed macrophage subpopulation in TAK aortic inflammatory lesions in comparison with atherosclerotic patients and heart transplant donors [41].

Consistent with the prevalent Th1 response in the pathophysiology of TAK, M1 macrophages were more frequent in the aorta of TAK and atherosclerotic disease patients compared to heart transplant donors. However, the expression of the M2 macrophage marker CD206 was higher than the M1 marker CD86 in the aorta of TAK patients [41]. This finding could be explained by the peculiar ability of IL-17A to induce a hybrid profile of macrophages with M1/M2 features, known as atypical M2-like macrophages with increased expression of CD206 [42].

In addition to IL-17-mediated polarization, other cytokines contribute to the phenotypic and functional shift of macrophages. Specifically, IFN-γ induces pro-inflammatory macrophages producing IL-1β, IL-6, and platelet-derived growth factor (PDGF), while Granulocyte-Macrophage Colony-Stimulating Factor (GM-CSF) triggers macrophages toward CD206^+^ and production of YKL-40 and MMP-9. Macrophage Colony-Stimulating Factor (M-CSF) also appears to be related to FRβ expression and PDGF release [39].

Recently, further cell subtyping in GCA identified the spatial distinction of CD206^+^/YKL-40^+^/MMP-9^+^ macrophages, engaged in tissue damage and situated in the media and damaged elastic lamina, and FRβ^+^/CD206^−^ macrophages resided in the adventitia and intima, responsible for wall fibrosis and intimal hyperplasia [43]. According to the aforementioned, the spatial gradient of GM-CSF and M-CSF would mirror the polarization of macrophages, which is markedly affected by the surrounding milieu [44].

Macrophages are thus corroborated as major players that participate both in the early phase of the disease, through the establishment of a systemic inflammatory environment, and in the advanced phase, where they take part in vascular remodeling by promoting angiogenesis, intimal hyperplasia, and tissue damage. In the latter function, macrophages are assisted by a plethora of molecules required to accomplish the structural changes in the arterial vessels leading to the major clinical complications. 

Among these, MMP exert their tissue-invasive capacity while facilitating the access of other inflammatory cells. In particular, MMP-2 and MMP-9 are mainly detected in GCA lesions adjacent to the internal elastic lamina, and MMP-9 is crucial in LVV as it allows monocytes and T cells access to the vascular wall through collagen IV membranes [45], [46].

IFN-γ-activated macrophages, giant cells, or injured vascular smooth muscle cells (VSMCs) produce growth factors, essentially PDGF and VEGF, that contribute to vessel wall remodeling [47]. 

Despite the already consolidated role of immune checkpoints in DC, macrophages also appear to be affected by the alteration of these pathways. As well as DC, macrophages from GCA patients express low PD-L1, in contrast to macrophages from patients with coronary artery disease (CAD) [48] 123047. Recently, another inhibitory immune checkpoint has been related to GCA in an elegant study where a defect in the CD155-CD96 checkpoint in GCA macrophages has been described [49]. Retention of the inhibitory ligand CD155 in the endoplasmic reticulum promoted APC dysfunction, resulting in the expansion of CD96^+^ memory CD4 T cells in the blood and inflamed arteries and the production of IL-9, a cytokine implicated in vascular remodeling and tissue-destructive immunity [40,50].

Taken together, these observations strengthen the active function of macrophages in orchestrating the coordinated network that promotes inflammatory cell distribution in arterial wall layers and the upregulation of relevant molecular pathways in LVV. 

## 4. Polymorphonuclear Leukocytes: An Heterogenous Group of Cells Deeply Involved in LVV Pathogenesis 

PMN leukocytes constitute the first line of defense against pathogens and potently drive innate immune inflammatory responses through the release of several pro-inflammatory mediators contained in cytoplasmic granules. PMN include neutrophils, eosinophils, basophils, and mast cells (MC). 

In LVV, basophils and eosinophils have been poorly described. Interestingly, although eosinophils’ role is emerging in the context of eosinophilic syndromes, including eosinophilic granulomatosis with polyangiitis (EGPA), their complex biology in other vasculitis is still far from being elucidated [51]. In ANCA-associated vasculitis (AAV), the damage is mainly mediated by eosinophilic cationic protein (ECP) and eosinophil-derived neurotoxin (EDN), and the presence of peripheral and tissue eosinophilia has been reported in AAV other than EGPA, in drug-induced vasculitis, and in Kawasaki disease [52,53,54]. Considering LVV, some case reports described the association of TAK or temporal arteritis with eosinophilic disease, presenting as peripheral hypereosinophilia or eosinophilic gastroenteritis [55,56]. 

However, since eosinophilia is not universally present in vasculitis other than EGPA, only few studies have focused on their possible activation in these conditions, making very difficult to establish a clear definition of their eventual pathogenic role. 

On the other hand, neutrophils and mast cells seem to play an essential role in vascular damage initiation and progression, and their aberrant functions will be deeply detailed in the following paragraphs. 

## 5. Mast Cells Display Pleiotropic Functions in LVV with a Possible Dichotomic Behavior in Boosting Inflammation

MC are unique, polifunctional innate immune cells of our immune system that have been recently depicted as active key players in autoimmunity [57]. MCs are widely distributed in tissues and participate in tissue homeostasis and repair [58]. MC exerts a pleiotropic role in arterial damage in LVV, as they can actively contribute to the initial inflammatory phase and the subsequent vascular remodeling which comprises both aberrant neo-angiogenesis and deregulated fibrosis, with pro-angiogenic and pro-fibrotic factors dominating the scene [59,60]. 

Upon activation, MC secrete pro-inflammatory mediators, like TNF-α, IL-6, IL-13, monocyte chemoattractant protein (MCP)-1, MCP-3, and macrophage inflammatory protein (MIP)-1α, that contribute to vascular disruption [57]. Simultaneously, MC are a major source of VEGF [61], a growth factor deeply involved in the deranged vascular remodeling accompanying inflammatory arterial damage, as demonstrated in abdominal aortic aneurysm, myocardial infarction, and atherosclerosis [59,62,63]. In LVV, MC are activated by IL-33, which is overexpressed in inflamed arteries and elicits VEGF production after binding its cognate receptor ST2 receptor, which was found to be markedly increased in MC infiltrating GCA and TAK specimens [64]. MC in the neointima of GCA temporal arteries were described, further supporting their proinflammatory role. Histologically, a spatial association between MC, neovessels, and T cells was evidenced, confirming the possible interaction between MC and T cells in driving vessel wall injury [65]. Interestingly, in control biopsies, MC were exclusively found in the adventitia, suggesting that their location in deeper arterial layers is intimately associated with wall-damage occurrence [66]. 

In TAK, MC display enhanced capabilities in determining permeability and angiogenesis, which are prevented by IL-33 inhibition [67,68]. However, MC seem to be strongly involved in the final act of vascular alteration in TAK, consisting of exuberant fibrosis, almost independent from IL-33 signaling. In 2022, elevated circulating markers of MC activation were demonstrated in TAK, and MC, cultured in vitro with sera derived from TAK patients, produced high levels of PDGF and TGF-β. Fibroblasts stimulated with the supernatant revealed an upregulation of markers of activation, namely collagen, fibronectin, and alpha-smooth muscle actin (α-SMA), outlining a prominent role of MC in the pathogenic pathways leading to wall fibrosis. IL-33 inhibitors did not affect the promotion of profibrotic phenotypes in fibroblasts, suggesting that unknown signaling pathways may be implicated in such a process [68]. 

Despite their proinflammatory functions, concurring in shaping inflammation and aberrant resolution in LVV, MC display potential immunoregulatory capabilities that make the scenario even more puzzling. In asthma, MC are continuously stimulated by IL-33 and lose their responsiveness. In this regard, IL-33 might then protect against chronic inflammation rather than perpetrate it [69]. In addition, MC control Treg homeostasis via IL-9 stimulation, and interestingly, such a molecule was reported as a possible driver of vessel inflammation [50]. So, IL-9 signaling may cooperate in acquiring peripheral tolerance in inflamed tissues. By the way, the exact role of Treg-MC cross-talk is still unclear in LVV but could suggest a beneficial function of MC in the vasculitis trajectory [70].

Probably, future studies considering the precise distribution in the injured arterial wall, cell-cell interaction, and functional commitment of MC in different disease phases will clarify the potential double-sided behavior and differential role of MC in LVV pathogenesis. 

## 6. Neutrophils: Old Cells with Newly Described Functions and Plasticity in Driving Both Inflammation and Fibrosis in LVV

**Figure 1:**
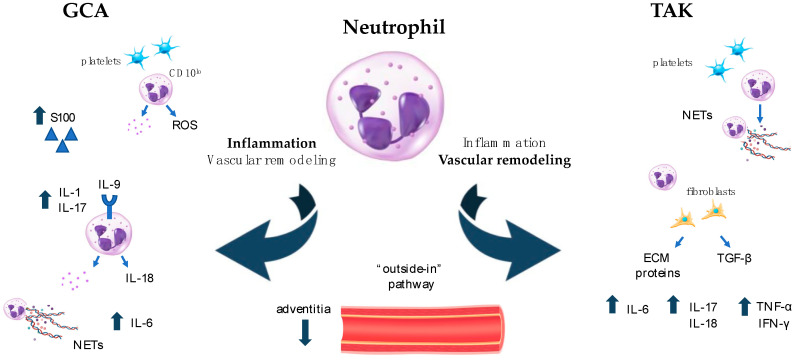
Neutrophil activation in LVV. Differential roles of neutrophils in LVV. In GCA (**left** side of Figure 1), neutrophils contribute to and sustain inflammation through the production of pro-inflammatory cytokines and chemokines, ROS, and NETs. They interact with platelets to drive immune-thrombosis and cooperate with the complement system in amplifying the inflammatory loop. In TAK (**right** side of Figure 1), neutrophils play a crucial role in vascular remodeling that follows the initial inflammatory burst and culminates with important fibrotic changes. They directly produce pro-fibrotic molecules and promote fibroblast differentiation, thus eliciting further production of ECM components. ECM: extracellular matrix; GCA: giant cell arteritis; IFN: interferon; IL: interleukin; LVV: large vessel vasculitis; NETs: neutrophil extracellular traps; ROS: radical oxygen species; TAK: takayasu arteritis; TGF: transforming growth factor; TNF: tumor necrosis factor.

The involvement of neutrophils in vasculitis pathogenesis is supported by the high neutrophil-to-lymphocyte ratio observed in the peripheral blood of patients. The dangerous arsenal of neutrophils consists of proinflammatory cytokines, ROS, proteolytic enzymes, and neutrophil extracellular traps (NETs) [71]. Moreover, neutrophils efficiently interact with immune cells as well as with wall-resident cells to shape the microenvironment typical of vascular inflammation [72]. The multifaced effects of neutrophils have been deeply characterized in small vessel vasculitis, particularly in AAV [73]. In contrast, neutrophils’ importance in LVV has been largely underestimated, with only a few studies shedding light on their active contribution in driving vascular damage in such diseases (Figure 1).

In particular, neutrophils seem to actively participate in both acute inflammatory and chronic fibrotic disease stages in LVV [74].

During the active phase of LVV, neutrophils can invade the arterial wall, transmigrating from the adventitial small vessels, thus contributing to the “outside-in” pathway of inflammation.

In support of this observation, neutrophils were described in vasa vasorum and small vessels surrounding temporal arteries in GCA, as well as in the aorta walls in TAK [75]. In the small vessels in the adventitia, neutrophils produce alarmins, such as S100 proteins, that can boost endothelial cell activation. Particularly, S100A12 is abundant in the adventitia of injured vessels, and its serum concentration is increased in GCA and correlates with disease activity, erythrocyte sedimentation rate (ESR), and C-reactive protein (CRP) levels [76]. Moreover, the proinflammatory milieu in GCA is enriched with IL-1β, IL-17, and IL-9, which promote granulopoiesis, neutrophil migration, and degranulation [22,77]. Notably, the IL-9 receptor is upregulated in neutrophils found in transmural and small vessel vasculitis GCA pathotypes. IL-9 drives the production of IL-8, whose levels are elevated in GCA, bridging IL-9 and neutrophil hyperactivity in inflamed vessels [50]. Even in TAK, IL-17, IL-8, IFN-γ, and TNF-α are markedly overexpressed and sustain neutrophil recruitment, activation, and survival under inflammatory conditions [78,79]. Among cytokines, IL-1β, TNF-α, and IL-18 were strongly related to neutrophil activation, as demonstrated in in vitro essays [80], making those molecules potential cues for the release of NETs during the systemic inflammation syndrome that characterizes LVV. Moreover, it has been shown that IL-6, produced by neutrophils infiltrating the adventitia of the inflamed aorta in TAK, results in progressive destruction of the arterial wall. Similarly, the expression of IL-6 by NETs and intact neutrophils infiltrating GCA temporal artery biopsies suggests that IL-6 may actively participate through neutrophils in the tissue inflammatory loop [81]. 

In a recent study, the role of NETs has been nicely outlined in LVV. In particular, the accumulation of NETs has been related to a double mechanism involving increased NET formation and simultaneous impaired NET degradation. The latter is due to the presence of anti-NET IgG antibodies that may abrogate the constitutive function of DNase to lyse NETs. Additionally, NETs-derived autoantigens may precipitate in immunocomplexes that further blunt NETs degradation and perpetrate the inflammatory loop [80]. In addition, NETs could be an active source of antigens, and in TAK, markedly increased titres of anti-histone antibodies were found. Their role is not yet understood, but they may participate in the inflammatory response in systemic vasculitis.

Finally, beyond cytokines, NETs may form after platelets-neutrophils interplay, for example via TLR9-dependent CXCL4 release, as demonstrated in AAV [82]. Consequent platelet aggregation on the NET scaffold activates the coagulation cascade and drives immune thrombosis [83]. In LVV, platelets display features of overactivation, as suggested by the increased concentration of TSP-1, and the association with NETosis supports a detrimental partnership between platelets and neutrophils in LVV development [80].

Further research detangling NETosis in LVV is awaited to define its role as a new disease driver and as a possible future target of tailored therapies [71]. 

The plasticity of neutrophils has been recently acknowledged by the identification of several phenotypes retrieved in peripheral blood in different stages of disease and under treatment. 

In particular, chronic neutrophilia in GCA, even observed under corticosteroid therapy, raised the hypothesis of an important role for neutrophils in GCA initiation and recurrence consistent with immunophenotype shifting. In particular, within the first week of therapy, neutrophils show a hyporesponsive phenotype (CD16^hi^AnxA1^hi^CD62L^lo^CD11b^lo^), while in contrast, at 6 months, they recover their activated status and deregulated capacity for binding to endothelial cells. At the same timepoint, the percentage of neutrophils able to suppress T-cell functions, identified as CD16^bright^CD62L^dim^CD11b^bright^ [84], is deeply decreased, paving the way for an escape pathway driving GCA relapse [85].

Recently, the presence of immature low-density neutrophils (CD10^lo^) in both blood and temporal tissue from GCA patients was highlighted. In particular, immature neutrophils escape apoptosis, interact with platelets, and release high amounts of ROS. ROS foster the release of neutrophil granules and sustain endothelial inflammation. In GCA temporal specimens, immature neutrophils occupy the lumen and the adventitia, making them possible initiators of the elastic lamina disruption. The pathogenic function of immature neutrophils in GCA is further corroborated by their absence in healthy tissue and their marked decrease after treatment [86]. 

Differently from the inflammatory pathway described so far, neutrophils seem to deeply contribute to the fibrotic pathways leading to profound vascular remodeling in TAK. The thickening of the arterial wall due to fibrosis characterizes adventitial changes in TAK and depends on the imbalance between extracellular matrix (ECM) formation and degradation. Fibroblasts drive the process, contributing to the reshaping of the arterial wall towards fibrotic scars [87]. In experimental models of vascular damage, neutrophils were able to interact with fibroblasts, enhancing their profibrotic phenotypes with the consequent production of ECM proteins and TGF-β [88]. Moreover, neutrophils were demonstrated to be active producers of type I collagen in a mouse model of renal fibrosis [89]. So, it is reasonable that neutrophils in TAK adventitia may directly acquire a profibrotic phenotype, which contributes to worsening the development of scarring lesions with the final loss of a clearly defined architectural wall structure [72].

In conclusion, the complete knowledge of neutrophil phenotypes and functions can help in the diagnosis of early vasculitis, potentially relapsing disease, and the final fibrosis, as well as the development of treatment resistance, constituting an important pathogenic feature along the whole disease course. 

## 7. Other Immune Pathways Involved in Innate Immunity: Focus on Complement System and Inflammasome Activation in LVV

### 7.1. Complement System

The complement system is a major component of innate immunity and, once triggered by any of its three activation pathways (classical, lectin, or alternative), displays various effector functions, including the induction of inflammation and cell damage [90]. 

The complement pathway has been implicated in the pathogenesis of vasculitis, although the underlying molecular mechanisms are unclear. 

Several studies conducted in recent years have corroborated the role of complement activation in ANCA vasculitis, particularly in renal involvement, where complement deposits have been observed in renal biopsies of MPO ANCA-positive and PR3 ANCA-positive patients. In particular, C5a has displayed a crucial role in ANCA-mediated lesions, acting as a stimulant for neutrophils with a positive feedback mechanism. Such evidence suggests the possibility of selectively inhibiting the C5a axis as a potential therapeutic option [91]. 

Another molecule of major interest in the pathogenesis of LVV is pentraxin 3 (PTX3), which not only contributes to the inflammation but also actively participates in the mechanism of vascular remodeling in TAK [92]. 

As evidenced, high PTX3 serum concentrations are associated with disease activity and high levels of acute-phase proteins. In addition, the further function of PTX3 in mediating C1q complement activation might suggest a possible pathogenetic role to be elucidated, considering that C1 activation and the presence of anti-C1 antibodies are featured in several rheumatologic diseases, including vasculitis [93,94,95]. 

Another approach to investigating the role of complement is to measure the levels of its activation products in patient serum. 

In this regard, increased serum levels of C3 and C4b have been detected in TAK patients and correlated with other acute-phase proteins, disease activity, and vascular stenosis progression (VST), assuming that serum C3 could be a potential biomarker for assessing disease activity in TAK [96]. 

In TAK, the presence of elevated autoantibody anti-epithelial cells (AECA) in the serum has been found to potentially mediate complement-dependent cytotoxicity, which contributes to the development of vascular pathogenic lesions. In addition, infectious antigens may trigger an acute inflammatory response by triggering the classical pathway of the complement system, leading to the activation of C3 [97].

Similarly, high levels of C4BP have been observed in patients with active TAK, as well as in other B-cell-mediated diseases such as systemic lupus erythematosus. Specifically, C4BP is a high-molecular-weight plasma glycoprotein synthesized in liver cells that is engaged in inhibiting the activation of classical and lectin complement pathways, modulating the anticoagulant activity of protein S, and supporting the survival of B lymphocytes. This is not surprising considering the recent growing interest in the potential role of B cells in the pathogenesis of TAK disease [98]. 

Therefore, in light of its multifaceted impact, the precise role of complement in the pathogenesis of LVV should be further explored.

### 7.2. Inflammasome 

Inflammasomes represent large cytosolic multiprotein complexes that play a crucial role in the immune reaction by assembling in response to infection- or stress-related stimuli. Inflammasomes trigger caspase-1-mediated inflammatory responses, leading to the cleavage and unconventional secretion of proinflammatory cytokines, such as IL-1β and IL-18, and initiating pyroptosis, an inflammatory form of cell death [99]. While inflammasomes are primarily associated with host defense against pathogens, recent studies suggest their critical role in various chronic diseases, including gout, atherosclerosis, metabolic syndrome, and autoimmune diseases. The nucleotide-binding oligomerization domain-like receptor (NLR) family, AIM2-like receptor (ALR) family, and RIG-I-like receptor (RLR) family are involved in nucleating inflammasome assembly. Among the NLR family, NLRP3, distinct for its broad recognition of diverse stimuli, plays a key role in sterile inflammatory diseases [100]. Notably, the NLRP3 inflammasome complex represents the first level of response to any endogenous and exogenous tissue damage, especially at the vascular level, as demonstrated by some evidence in aortic pathologies like aneurysms and dissections [101]. The chronic inflammatory background mediated by NLRP3/IL-1 acts as a link between metabolic stresses and innate immune sensors, eliciting vascular inflammation [102]. This resemblance is observed in vasculitis and autoinflammatory diseases and is associated with endothelial dysfunction.

A recent study revealed that systemic inflammation relies on the NLRP3 inflammasome’s activity, which is under the inhibitory control of cAMP-dependent molecule-specific selective autophagy. This process involves the activation of MARCH7, an E3 ubiquitin ligase [103]. Additionally, tripartite motif proteins function as specialized receptors facilitating precision autophagy, specifically targeting crucial inflammasome components. This interplay underscores the intricate regulation of inflammasome function by autophagy mechanisms, shedding light on potential therapeutic avenues for modulating inflammatory responses [104]. The above-mentioned common pathogenic pathways are shared between systemic autoimmune diseases and atherosclerosis. 

Despite the potential magnitude of inflammasome research, very few studies have analyzed the possible role of inflammasomes in LVV. 

Genome-wide association studies have identified significant associations between TAK in Japanese patients and single-nucleotide polymorphisms in the MLX gene, which encodes the MLX (Max-like protein X) transcription factor. A recent investigation [105] focused on the MLX single-nucleotide polymorphism rs665268, a missense mutation causing the Q139R substitution in MLXs DNA-binding site. This mutation demonstrated a notable correlation with Takayasu arteritis severity, including the number of arterial lesions and aortic regurgitation morbidity. The heightened activity of the NLRP3 inflammasome and increased cellular oxidative stress were identified as contributing factors. Since IL-1β is a major product of the NLRP3 inflammasome and a mediator of inflammatory responses, th study examined whether the MLX-Q139R mutation influenced IL-1β production [106]. Results from measuring IL-1β levels in supernatants of cultured peripheral blood mononuclear cells (PBMC)-derived macrophages using ELISA indicated significant upregulation of IL-1β secretion in PBMCs from TAK patients carrying the risk genotype compared to normal subjects. Interestingly, no significant differences were observed between the two groups in the secretion of other major proinflammatory cytokines, TNF-α and IL-6. In summary, these findings suggest that the MLX-Q139R mutation promotes NLRP3 inflammasome formation, leading to increased IL-1β production.

The assembly of the inflammasome, essential for activating caspases 1 and 5 and processing various inflammatory cytokines, relies on the scaffold provided by Low-Density Lipoprotein Receptor-Related Protein 1 (LRP1). This discovery identifies LRP1 as a novel susceptibility gene for GCA, indicating a potential dysregulation in the recruitment of the inflammasome in large vessel vasculitis. This insight suggests that LRP1 could serve as a therapeutic target in the context of GCA. Moreover, in a recent study, a genetic association of NLRP1 with GCA was found by genotyping a single-nucleotide polymorphism (rs8182352), which has been reported to confer risk to the development of autoimmune processes in previous studies [107].

There is a need for further research to elucidate specific signals leading to the assembly of different inflammasomes. While the focus has been on infectious triggers, nonmicrobial stimuli activating NLRP3 and the potential roles of other NLRs in sensing nonmicrobial signals of physiological stress remain areas for exploration.

## 8. Conclusions and Future Therapeutic Perspectives

LVV are autoimmune and autoinflammatory diseases, and the underlying complexity of their pathophysiology reflects the challenge of achieving personalized treatments. Knowledge about the role of the innate immune response in LVV has improved dramatically in recent years, leading to the discovery of new therapeutic targets to optimize patient treatment and reduce the use of glucocorticoids.

Besides, the validated use of tocilizumab, a humanized monoclonal antibody targeting the IL-6 receptor, in the treatment of LVV [108,109], other potential therapeutic avenues of interference with disease-relevant innate immunity pathways are set to reshape the next scenario.

In this regard, GM-CSF is a multifunctional cytokine that can modulate DC, macrophages, and CD4^+^ T lymphocytes; blockade of the GM-CSF receptor in cultured temporal arteries resulted in decreased transcription of Th1 and Th17-associated genes (e.g., IFN-γ and IL-6). In a phase 2 randomized, double-blind, placebo-controlled trial, Mavrilimumab, a monoclonal antibody that blocks GM-CSF signaling, significantly reduced the risk of flare and improved sustained remission rates compared with placebo with a 26-week taper of prednisone in patients with GCA [110].

Moreover, targeting macrophage-associated molecules holds promise for controlling vascular remodeling in LVV, such as blocking MMP-9 or using anti-angiogenic therapies [46,111].

The molecular signaling cascade and pathways promoting neutrophil-mediated inflammation deserve to be further elucidated in LVV. In particular, mechanisms leading to NET generation may unravel new targets for pharmaceutical interventions in the future. Drugs targeting the complement system or endopeptidase able to cleave autoantibodies in the Fc portion appear as suitable options to dampen neutrophil activation. Additionally, nanoparticles are currently under investigation as possible effective treatment strategies to reduce neutrophil and macrophage inflammatory activity [72].

Recently, clinical efficacy against atherosclerotic and inflammatory vascular diseases has been demonstrated by the development of molecularly targeted drugs that inhibit each stage of the pro-inflammatory NLRP3 inflammasome/IL-1β/IL-6 pathway [112]. Overall, understanding inflammasome activation mechanisms and their implications for host defense and vascular inflammation is critical for advancing therapeutic interventions.

Various drugs modulate the inflammasome, with colchicine limiting caspase-1 activation elicited by the inflammasome [113] and IFN-α exhibiting a dual inhibitory effect on the inflammasome [114]. Targeting the NLRP3 inflammasome-driven IL-1β holds promise as a therapeutic approach for patients with atherosclerosis and sterile inflammatory diseases. Presently, no approved selective inhibitor specifically targeting the NLRP3 inflammasome exists for clinical application. Instead, IL-1-targeting agents like anakinra (recombinant IL-1Ra), rilonacept (a soluble decoy receptor for IL-1), and canakinumab (a humanized monoclonal IL-1β antibody) are employed [115] in treating patients with cryopyrin-associated periodic syndrome, characterized by a gain-of-function mutation in the NLRP3 gene encoding cryopyrin, as well as other autoinflammatory diseases [100]. Finally, JAK inhibition, which has a broader effect on intracellular signaling by impacting both components of innate immunity as well as T-cell activation, seems promising as a strategy for the treatment of LVV [116]. In particular, given the known role of IL-6, type I IFN, and GM-CSF in LVV, all of which use the JAK-STAT pathway, the application of JAK inhibitors could have a strong rationale for controlling vascular inflammation. Up to date, most of the data on the efficacy of JAK inhibitors in LVV are derived from case reports [117,118]. However, clinical trials of JAK inhibition for GCA and TAK are ongoing [119].

In light of the limited data regarding the role of innate immunity cells in LVV, predominantly derived from in vitro or ex vivo experiments and histological findings, longitudinal studies aimed at investigating changes in these cells after treatment would be useful. The application of “omics sciences” (proteomics, transcriptomics, and metabolomics) could also be an additional approach for the study of such cell subsets, often marked by low abundance and closely influenced by the surrounding milieu, as well as for the in-depth investigation of the link between inflammation and metabolites.

Therefore, considering the puzzling and intricate immunological and molecular network underlying vascular inflammation, knowledge of the role of innate immunity cell subpopulations in the pathogenesis of LVV and their phenotypic variability can help us better define the clinical spectrum of the disease and guide future targeted therapies from a precision medicine perspective.

## Figures and Tables

**Table 1:** Main features of innate immunity cells in LVV.

	GCA	TAK
Mast cells		
	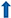 VEGF-ST2 receptor Presence in the neointima	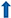 PDGF and TGF-β Wall fibrosis
Neutrophils		
	Vasa vasorum and small vessels in the temporal arteries 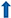 S100A12, IL-1β, IL-17, IL-9, IL-8, IL-6	Aorta walls; 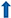 IL-17, IL-8, IFN-γ, TNF-α 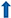 Anti-histone antibodies Wall fibrosis
	NET activation Partnership with platelet activation	
Macrophages		
	M1 in adventitia and media M2 in the media-intima border CD206+/YKL-40+/MMP-9+ in media FRβ+/CD206- in adventitia and intima 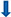 PD-L1 expression 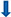 CD155-CD96 checkpoint	All layers of the vessel M1 is more frequent in the aorta
Dendritic cells (vasDCs)		
	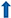 Expression of CCR7, CCL19 and CCL21 TLR4 and TLR5 ligands promote vascular damage 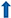 IL-6, IL-18, IL-23, IL-32, and IL-33 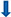 PD-L1 expression	T cells co-localize with DC in the adventitia of the aortic wall The involvement of DC remains marginal
Complement		
	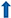 C3, C4b, and C4BP correlate with disease activity and vascular stenosis progression	
Inflammasome		
	Genetic association of NLRP1 with GCA was found genotypinge a single-nucleotide polymorphism (rs8182352)	MLX-Q139R mutation promotes NLRP3 inflammasome formation, leading to increased IL-1β production

DC: dendritic cells; GCA: giant cell arteritis; IFN: interferon; IL: interleukin; LVV: large-vessel vasculitis; MLX: Max-like protein X; MMP: matrix metalloproteinases; NET: neutrophil extracellular traps; NLRP1: nucleotide-binding oligomerization domain-like receptor;PDGF: platelet-derived growth factor; PD-L1: programmed cell death protein -ligand 1; TAK: Takayasu arteritis; TGF: transforming growth factor; TLR: toll-like receptor; TNF: tumor necrosis factor; VEGF: vascular endothelial growth factor.
